# Interleukin-27 and Its Diverse Effects on Bacterial Infections

**DOI:** 10.3389/fimmu.2021.678515

**Published:** 2021-05-17

**Authors:** Yugo Morita, Elysia A. Masters, Edward M. Schwarz, Gowrishankar Muthukrishnan

**Affiliations:** ^1^ Center for Musculoskeletal Research, University of Rochester Medical Center, Rochester, NY, United States; ^2^ Department of Orthopaedics and Rehabilitation, University of Rochester Medical Center, Rochester, NY, United States; ^3^ Department of Biomedical Engineering, University of Rochester Medical Center, Rochester, NY, United States

**Keywords:** cytokine signaling, bacteria, sepsis, bacterial infection, IL-27 cytokine

## Abstract

Innate and adaptive immune responses against pathogens are known to be carefully orchestrated by specific cytokines that initiate and down regulate immune cell functions from the initial infection through tissue repair and homeostasis. However, some cytokines, including interleukin-27, are expressed at multiple phases of the infection, such that their pro and anti-inflammatory functions have been difficult to interpret. As elucidation of specific cytokine functions throughout infection is central to our understanding of protective *vs.* susceptible immunity and return to homeostasis *vs.* prolonged inflammation leading to septic shock, here we review the literature on IL-27 signaling and the various functions of this heterodimeric ligand member of the IL-12 cytokine family. Canonically, IL-27 is produced by antigen-presenting cells, and is thought of as an immunostimulatory cytokine due to its capacity to induce Th1 differentiation. However, many studies have also identified various immunosuppressive effects of IL-27 signaling, including suppression of Th17 differentiation and induction of co-inhibitory receptors on T cells. Thus, the exact role of IL-27 in the context of infectious diseases remains a topic of debate and active research. Additionally, as recent interest has focused on clinical management of acute *vs.* chronic infections, and life-threatening “cytokine storm” from sepsis, we propose a hypothetical model to explain the biphasic role of IL-27 during the early and late phases of immune responses to reconcile its known pro and anti-inflammatory functions, which could be therapeutically regulated to improve patient outcomes of infection.

## Introduction

Interleukin (IL) -27 is a cytokine with remarkably diverse influences on the immune system (1, 2). IL-27 is composed of IL-27p28 and Epstein-Barr virus–induced 3 (EBI3) subunits, and signals through a heterodimeric cell surface receptor composed of IL-27Rα and gp130 (1). IL-27 is a unique cytokine with reported immunostimulatory and immunosuppressive effects on various immune cells (1, 3–17), and understanding of its precise role during bacterial infections remains incomplete. Prior studies using a murine sepsis model induced by subcutaneously injected *Escherichia coli* demonstrated that IL-27 blockade improves the survival rate (18). Similarly, IL-27 neutralizing antibody treatment reduced pulmonary inflammation and improved mice survival rate in a mouse model of cecal ligation and puncture (CLP)-induced acute lung injury (19). IL-27 inhibition in a murine model of secondary *Staphylococcus aureus* pneumonia following influenza infection also improved bacterial clearance (20). In contrast, blockade of IL-27 in rodent models of *Clostridioides difficile* infectious colitis decreased bacterial clearance and host survival rates (21). Furthermore, IL-27 has been shown to be critical to vaccine-elicited T cell responses (22). Therefore, the purpose of this review is to summarize the immunobiology of IL-27 in the context of bacterial infections and propose hypothetical mechanisms of IL-27-mediated immune homeostasis during these infections.

## Overview of IL-27 Components, Receptors, Signaling Pathways

**Figure 1:**
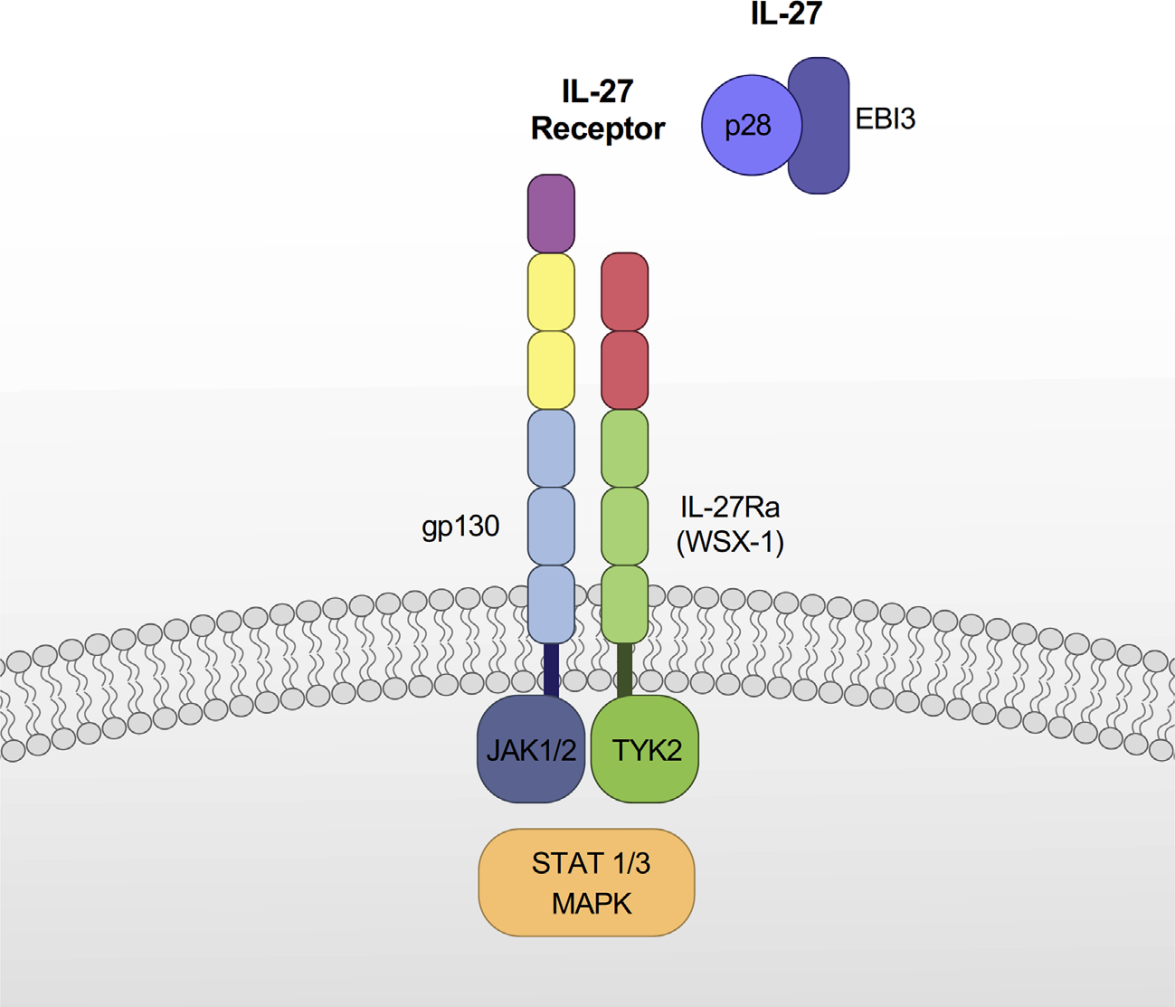
Components of IL-27 and IL-27 receptor. IL-27 is a heterodimeric cytokine composed of IL-27p28 and EBI3. The IL-27 receptor is a heterodimeric cell surface receptor composed of IL-27Rα and gp130. IL-27 signaling occurs primarily through a JAK/STAT pathway, which varies depending on immune cell types. IL-27 also signals through MAPK pathways.

IL-27 is a heterodimeric cytokine composed of IL-27p28 and EBI3 subunits (1) (**Figure 1**). EBI3 was originally identified as a soluble hematopoietin component related to IL-12p40 preferentially expressed in Epstein Barr virus-transformed B cells (23). In silico searches of orphan proteins that can bind to EBI3 led to the identification of IL-27p28, a four-α helical polypeptide bundle of the IL-6 cytokine family (1). IL-27p28 can bind to Cytokine-Like Factor-1 (CLF-1), and IL-27p28/CLF-1 complex enhances IFN-γ production in activated NK cells, inhibits CD4^+^ T cell proliferation to enhance IL-10 secretion (24). IL-27p28 is secreted as a soluble cytokine (called IL-30) by itself in mice, however, in humans, it is secreted as a heterodimer with EBI3. Muller et al. showed that the absence of a disulfide bond-forming cysteine pair in IL-27p28 determines whether it is secreted as a monomeric cytokine or if it is secreted in concert with EBI3 (25). In addition to IL-27p28, EBI3 can bind to IL-12p35 to form IL-35 cytokine, which exhibits immunosuppressive functions through the inhibition of T helper cell type 17 (Th17) differentiation and the promotion of regulatory T (Treg) cell proliferation (26, 27)

IL-27 signals through a heterodimeric cell surface receptor composed of IL-27Rα (also known as TCCR or WSX-1) and gp130 (5) (**Figure 1**). The interacting partner of IL-27Rα is the gp130 receptor, which is also utilized by several other cytokines for signaling, including IL-6, IL-11, leukemia inhibitory factor (LIF), oncostatin M (OSM), cardiotrophin 1 (CT-1), and ciliary neurotrophic factor (CNTF) (28, 29). Both IL-27Rα and gp130 are differentially expressed in numerous cells including monocytes, dendritic cells (DC), T and B lymphocytes, NK cells, mast cells, and endothelial cells (5). The expression levels of these receptors are also different depending on the activated state of cells (30, 31), which contributes to their altered responsiveness to IL-27 (31). For instance, naïve CD8^+^ T cells express gp130 and are highly responsive to IL-27, whereas CD8^+^ memory T cells are unresponsive to IL-27 due to decreased gp130 expression (31).

IL-27 signaling occurs primarily through the Janus kinase (JAK) - signal transducers and activators of transcription (STAT) pathway, with variations depending on immune cell types: mast cells (STAT3) (5), monocytes (STAT1, STAT3, and NF-κB) (32), macrophages (STAT1 and STAT3) (33), naïve CD4^+^ T cells (TYK2, JAK1, KAK2, STAT1, STAT2, STAT3, STAT4, and STAT5) (34) and naïve B cells (STAT1 and STAT3) (35).

**Figure 2:**
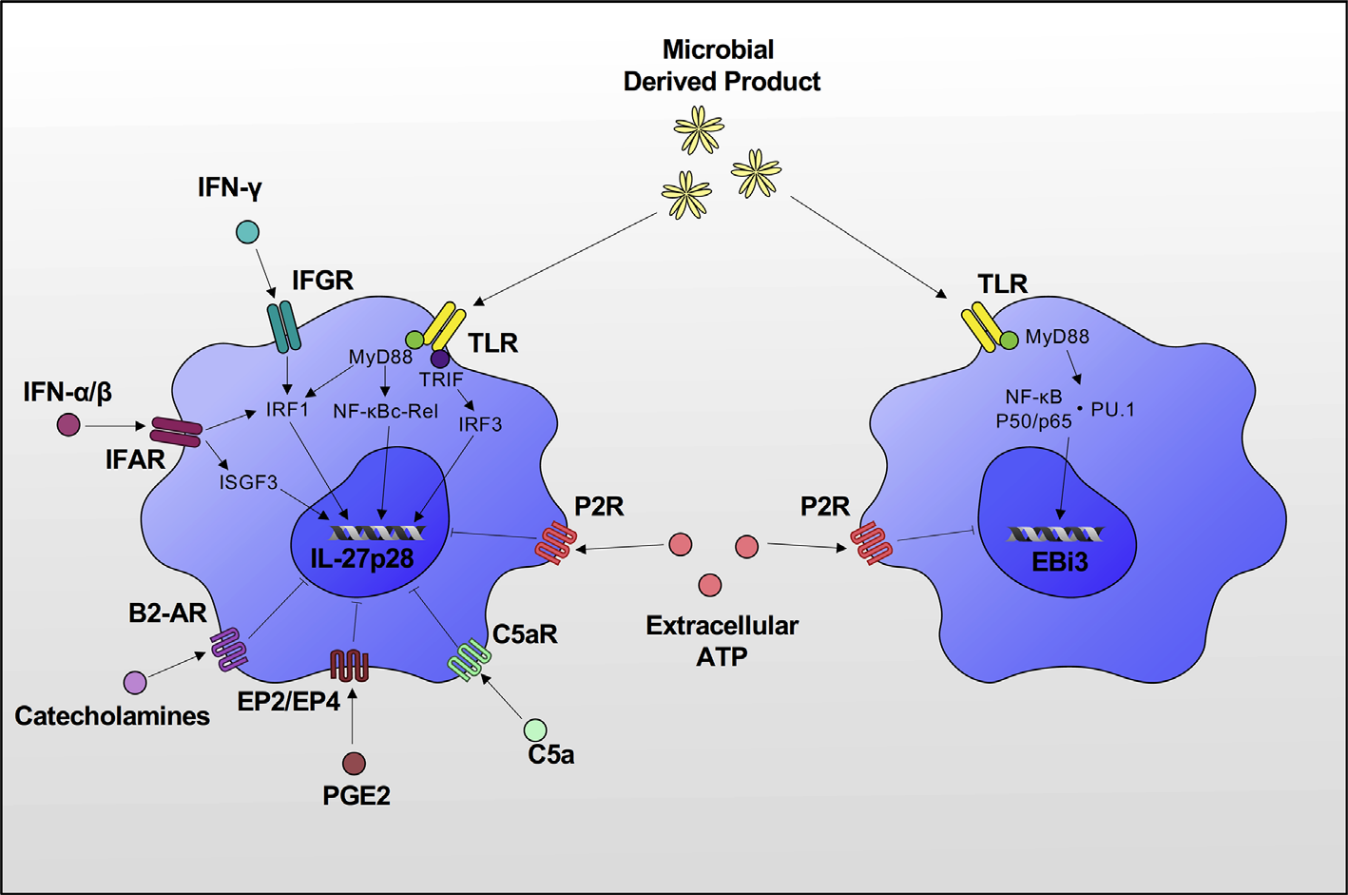
Induction of IL-27p28 & EBI3 expression. TLR stimulation increases expression of IL-27p28 through MyD88/NF-κB c-Rel, MyD88/IRF1 and TRIF/IRF3 signaling pathways. IFN-α/β/γ also upregulates IL-27p28 expression by activating IRF1. IFN-α/β is required for sustained expression of IL-27p28 *via* activation of IRF1 and formation of ISGF3 complex. Upregulation of EBI3 is also induced by TLR stimulation *via* activation of NF-κB p50/p65 and PU.1. There are also several inhibitors of IL-27 production. C5a, PGE2 and catecholamines inhibit IL-27p28 expression, and extracellular ATP inhibits both IL-27p28 and EBI3 expression *via* P2 receptor signaling.

IL-27 is mainly produced by antigen-presenting cells such as monocytes, macrophages and dendritic cells (1, 36–38). Other cell types including myeloid-derived suppressor cells (39), CD4^+^ and CD8^+^ T cells (40), osteoclasts (41), and activated B cells (42) are known to secrete varying levels of IL-27 as well. Microbial stimulation of Toll-like receptor (TLR) promotes the expression of IL-27 in these cells (37, 43, 44). For instance, TLR4 stimulation by lipopolysaccharide (LPS) increases expression of IL-27p28 through myeloid differentiation factor 88 (MyD88)/NF-κB c-Rel, MyD88/IFN regulatory factor (IRF)1, and Toll/IL-1R-related domain-containing adaptor-inducing IFN (TRIF)/IRF3 signaling pathways (37, 43, 45) (**Figure 2**). Type I and II IFNs also upregulate IL-27p28 expression by activating IRF1 (36–38), and type I IFN is required for sustained expression of IL-27p28 *via* activation of STAT1/IRF1 and formation of IFN-stimulated gene factor 3 (ISGF3) complex (45). The upregulation of EBI3 is induced by TLR stimulation *via* activation of NF-κB p50/p65 and PU.1 (46).

Several inhibitory factors of IL-27p28/EBI3 production have been reported including extracellular ATP that acts on the purinergic receptor of DCs (47) and C5a/C5aR on LPS-stimulated macrophages (48). A recent study demonstrated that prostaglandin E2 binds to the EP2/EP4 receptor and inhibits IL-27 production by downregulating IL-27p28 expression through the cAMP/IRF1 signaling pathway (49). The autonomic nervous system is also involved in the regulation of IL-27 production. Catecholamines *via* β2 adrenoceptor activation antagonize phosphorylation of JNK and suppress IL-27p28 production in LPS-stimulated macrophages (50) (**Figure 2**).

## Effect of IL-27 on Innate Immune System

### Monocytes, Macrophages, and Dendritic Cells

**Figure 3:**
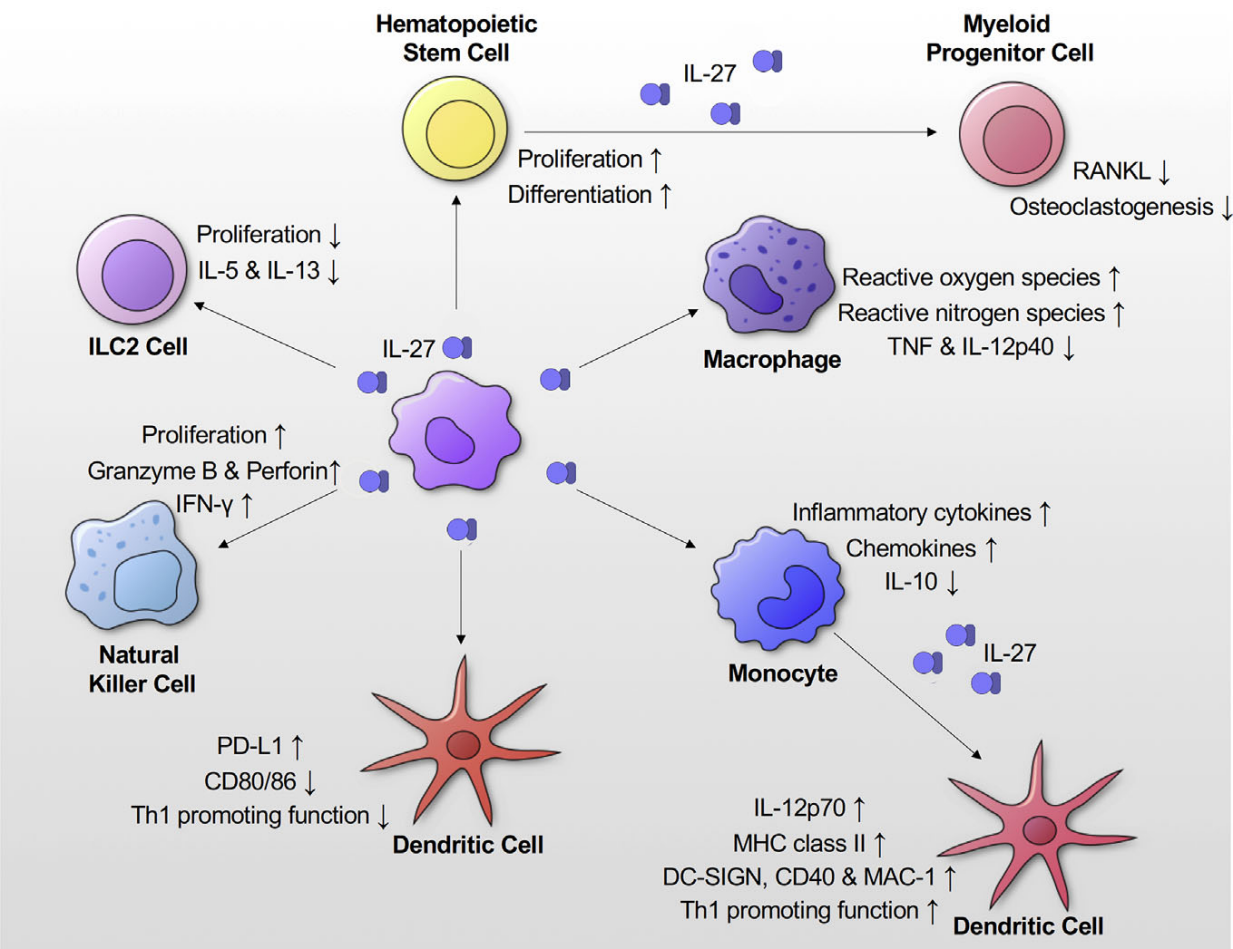
Effects of IL-27 signaling on innate immune responses. IL-27 signaling promotes proliferation and differentiation of hematopoietic stem cells. It also suppresses osteoclastogenesis. IL-27 signaling suppresses Th2 cytokines (IL-5 and IL-13) production and proliferation of ILC2 cells. IL-27 in concert with IL-15/IL-18 enhances proliferation of NK cells and production of IFN-γ, granzyme B and perforin by them. IL-27 promotes production of inflammatory cytokines and chemokines by monocytes. When DCs are differentiated from monocytes in the presence of IL-27 they show higher expression of MHC class II, DC-SIGN, CD40 and MAC-1. They also have increased production of IL-12. IL-27 enhances expression of PD-L1 on DCs, which show a reduced proliferation capacity and IFN-γ production.

IL-27 is acknowledged to have pro-inflammatory effects on monocytes, macrophages, and DCs (**Figure 3**). IL-27 stimulation alone leads to phosphorylation of STAT 1, STAT3 and enhances expression of inflammatory cytokines (IL-1β, TNF-α, IL-12p35, and IL-18) and chemokines (IP-10, MIP-1α, and MIP-1β) in primary human monocytes (5, 32). TLR7 and TLR8 activate different signaling cascades in monocytes, leading to distinct cytokine production and influence IL-27 expression in monocytes (51, 52). TLR8 increases expression of IL-27 in human monocytes, whereas TLR7 doesn’t directly increase IL-27 expression *via* induction of the transcription factor FOSL1 (52). However, several studies have demonstrated that TLR7/8 agonist resiquimod (R848) is a potent inhibitor of Th2 cell-driven inflammatory responses, which ultimately suppresses IL-27 in monocytes and macrophages (53–55). In addition, IL-27 increases TLR4 and TLR5 expression in THP-1 cells and phorbol 12-myristate 13-acetate (PMA)-stimulated THP-1 cells (17). IL-27 also acts as a costimulatory molecule with LPS, causing enhanced production of inflammatory cytokines (IL-12p40, TNF-α, and IL-6) in human peripheral blood mononuclear cells (PBMC), primary human monocytes, THP-1 cells, and PMA differentiated THP-1 cells (17). Similarly, Kalliolias et al. observed that primary human monocytes pretreated with IL-27, upon LPS stimulation, showed a marked increase in inflammatory TNF-α, IL-6 production, and a decrease in immunosuppressive IL-10 (10).

Nitric oxide (NO) is a source of reactive nitrogen species, which is important for killing of intracellular pathogens. Addition of IL-27 increases inducible NO synthase (iNOS) expression, and NO production in LPS stimulated mouse peritoneal macrophages. This process is signaled *via* activation of STAT1, p38 mitogen-activated protein kinase (MAPK), and NF-κB (13). Reactive oxygen species (ROS) is also crucial for eradicating intracellular pathogens, and IL-27 can enhance ROS production in primary human macrophages and dendritic cells upon PMA stimulation (56).

IL-27 also affects antigen-presentation function. In THP-1 cells, IL-27 stimulation causes increased expression of major histocompatibility complex (MHC) class I and II molecules, along with costimulatory CD80/86 and CD54, which aid in antigen presentation to immune cells (57). Primary human DCs differentiated in the presence of IL-27 exhibited two-fold higher expression levels of MHC II during *Staphylococcus aureus* infection. Increased IL-12 production was also observed in these cells, in addition to surface expression of dendritic cell-specific intercellular adhesion molecule-3-grabbing non-integrin (DC-SIGN), CD40, and macrophage-1 antigen (MAC-1), all of which leads to enhanced T cell proliferation and activation (14).

The aforementioned studies highlight the immunostimulatory activity of IL-27. In contrast, several studies have also elucidated the immunosuppressive effects of IL-27 on antigen-presenting DCs and macrophages. IL-27 treatment decreased TNF and IL-12p40 production in murine peritoneal macrophages upon IFN-γ and LPS stimulation or *Mycobacterium tuberculosis* infection (6). Similarly, IL-27 suppressed IL-12p40 production in LPS-stimulated murine bone marrow derived macrophages (58). Kalliolias et al. showed that IL-27 enhanced the production of proinflammatory cytokines (IL-1β, IL-6 and TNF-α) in human macrophages upon various TLR ligands stimulation. Interestingly, they also demonstrated that IL-27 suppressed responses of human macrophages to IL-1β and TNF-α *via* downregulation of their receptor expression on macrophages (59). Karakhanova et al. showed that human DCs treated with IL-27 enhanced programmed death-ligand 1 (PD-L1) surface expression leading to a reduced capacity to stimulate proliferation of DCs and IFN-γ producing T cells (11). Similarly, murine DCs from IL27Rα^-/-^ mice showed enhanced expression of costimulatory CD80/86 upon LPS stimulation leading to increased proliferation of IFN-γ producing T cells (9). Mascanfroni et al. also demonstrated that IL-27 signaling in murine DCs suppressed the generation of Th1 and Th17 cells *via* induction of the immunoregulatory molecule CD39, which depleted extracellular ATP and down-regulated inflammasome activation (60). In summary, through the many studies discussed here, we can reasonably conclude that IL-27 has predominantly elicited pro-inflammatory effects on monocytes. However, this is not necessarily the case with other antigen presenting cells such as DCs and macrophages. Nonetheless, further studies are necessary to clarify IL-27’s immunomodulatory role in these cells **(** **Figure 3** **)**.

### Innate Lymphoid Cells

Innate lymphoid cells (ILC) have recently been recognized as innate immune effector cells that are derived from the common lymphoid progenitors (61). Unlike, other innate cells and antigen presenting DCs, these cells: 1) lack phenotypic myeloid makers, 2) don’t have the typical lymphoid morphology, and 3) lack recombination activating gene (RAG)-dependent rearranged antigen receptors, typically found on B and T cells (62). ILCs have been classified based on functional criteria. One of group 1 ILCs, also known as natural killer (NK) cells, plays an important role in immune system regulation through IFN-γ production and cytotoxicity (62). IL-27, alone or in concert with other DC-derived cytokines (IL-15 and IL-18), has been reported to have a pro-inflammatory effect on NK cells (15, 63, 64). IL-27 stimulation increased IFN-γ production in primary human NK cells thorough activation of STAT1 and promoted activation of NK cells (upregulation of CD25 and CD69) (15). More recently, it was demonstrated that IL-27 treatment in concert with IL15/IL-18 enhanced proliferation of human NK cells and production of IFN-γ, granzyme B, and perforin (63). Group 2 ILCs (ILC2) that produce type 2 cytokines (IL-4, IL-5, and IL-13) play an important role in helminth infections and allergic diseases (61). A recent study showed that IL-27 treatment suppressed type II cytokine production and proliferation of murine ILC2 cells during lung inflammation induced by *Alternaria alternata*, a major fungus associated with ILC2-mediated asthma (65) **(** **Figure 3** **)**. These studies suggest that IL-27 is important for upregulation of type 1 cytokine responses in Group 1 ILCs and negative feedback mechanisms for type 2 innate pro-inflammatory responses. Further studies are needed to investigate IL-27’s role in regulating Group 3 ILCs.

## Effect of IL-27 on Adaptive Immune System

### CD4^+^ T Cells

**Figure 4:**
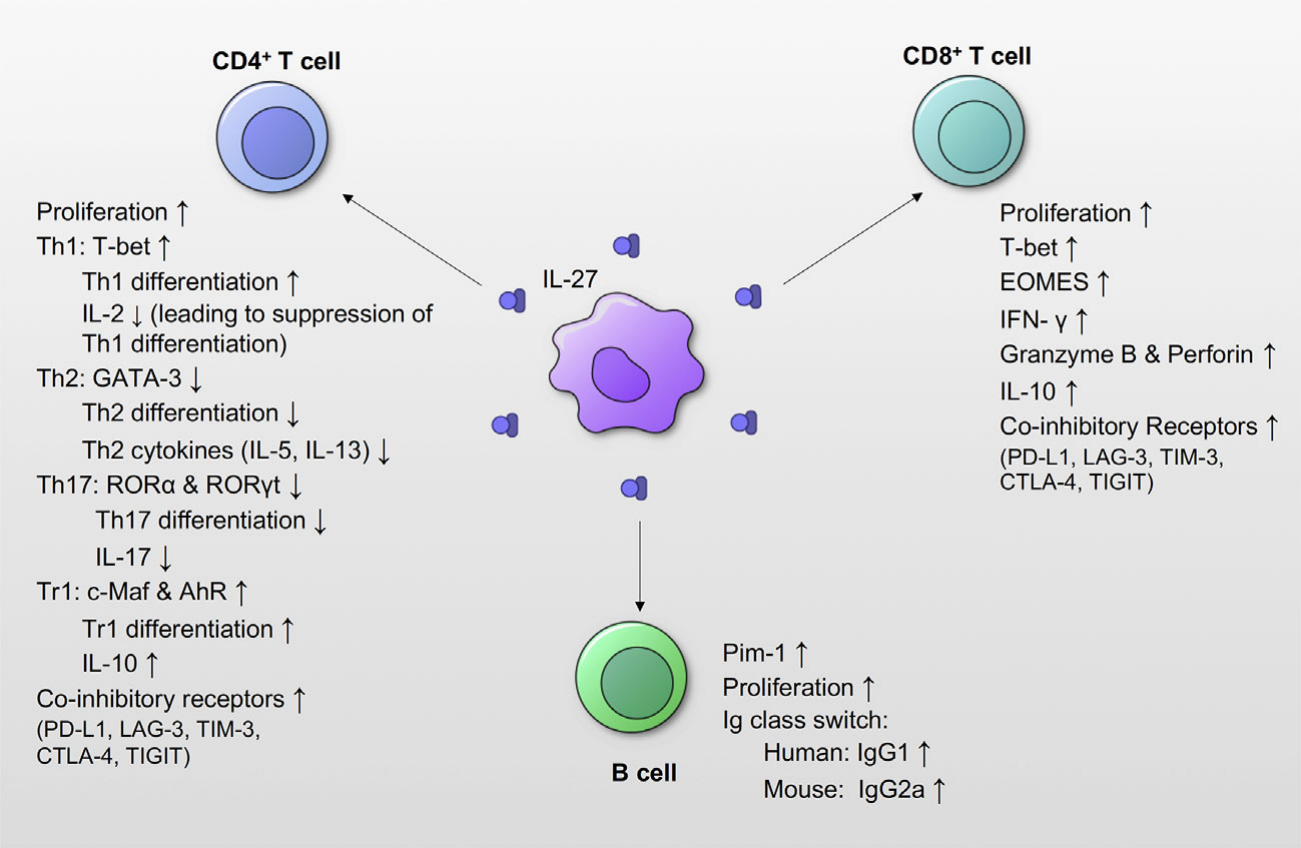
Effects of IL-27 signaling on adaptive immune responses. IL-27 signaling promotes proliferation of CD4^+^ T cells, CD8^+^ T cells and B cells. IL-27 promotes expression of the T-bet, and induces Th1 differentiation by increasing the expression of IL-12Rβ. However, subsequent IL-27signaling suppresses the production of IL-2, which leads to suppression of Th1 differentiation. IL-27 also suppresses the transcription factors GATA-3 and ROR, as well as Th2 and Th17 differentiation and function. IL-27 signaling induces Tr1 differentiation from T cells, and increases IL-10 production. IL-27 induces co-inhibitory receptors, and suppresses activated effector T cells. IL-27 also increases the expression of transcription factors T-bet and EOMES in CD8^+^ T cells, and increases production of IFN-γ, granzyme B and perforin. IL-27 also increases the production of IL-10 in CD8^+^ T cells. IL-27 increases the expression of Pim-1, which is a gene associated with cell proliferation and survival in B cells. IL-27 also induces IgG class switching. In humans, IL-27 increases IgG1, and IL-27 increased IgG2a in mice.

IL-27 has diverse effects on T cell proliferation, differentiation, and activation (2, 66) **(** **Figure 4** **)**. It promotes proliferation of naïve CD4^+^ T cells, and together with IL-12, mediates Th1 differentiation and IFN-γ production in naïve CD4^+^ T cells (1, 4). Stimulation of CD4^+^ naïve T cells by IL-27 promotes upregulation of Th1-specific transcription factor T-bet and IL-12Rβ2, both of which are essential for IL-12-mediated Th1 differentiation (3), suggesting a positive regulatory role in IL-27’s responsiveness to naïve CD4^+^ T cells. Interestingly, IL-27 also induces Th1 differentiation using T-bet-independent mechanisms *via* intercellular adhesion molecule (ICAM)-1/lymphocyte function-associated antigen (LFA)-1/extracellular signal-regulated kinase (ERK)1/2-dependent signaling pathways (67). On the other hand, IL-27 negatively regulates T cell growth and survival by affecting IL-2 production by CD4^+^ T cells through the expression of suppressor of cytokine signaling (SOCS) 3 (7, 8). These studies suggest that temporal changes in IL-27 production can both positively and negatively regulate Th1 development.

IL-27 suppresses Th2 cell differentiation by suppressing the expression of GATA-3, a transcription factor essential for Th2 response (3, 68). Further, IL-27 suppresses Th2 cytokine (IL-5 and IL-13) production from differentiated Th2 cells by diminishing GATA-3 expression (68). IL-27 also suppresses Th-17 responses by inhibiting IL-17, an essential inducer of pro-inflammatory cytokines and chemokines leading to migration of neutrophils (69–73). IL-27 downregulates the expression of Th17-specific transcription factor retinoid-related orphan receptor (ROR)α, RORγt, and inhibits IL-17 production from CD4^+^ T cells (70).

IL-27 limits proliferative T cell responses during infections and in autoimmune conditions to limit tissue damage (74, 75). IL-27 achieves this by inducing expression of co-inhibitory receptors such as programmed death-ligand 1 (PD-L1), lymphocyte-activation gene 3 (LAG-3), T cell immunoglobulin and mucin-domain containing-3 (TIM-3), cytotoxic T-lymphocyte-associated protein 4 (CTLA-4), and T-cell immunoreceptor with Ig and ITIM domains (TIGIT) on T cells (12, 16, 76). PD-L1 binding to Programmed cell death protein 1 (PD-1) negatively regulates T cell expression and suppresses its function by binding to CD80 as a co-inhibitory receptor (77, 78). IL-27 priming of naïve T cells induces PD-L1 upregulation in a STAT1-dependent manner, which inhibited Th17 differentiation in *trans* (12). IL-27 stimulation of Foxp3-expressing regulatory T cells (Tregs) induces expression of LAG-3, which enhances Treg suppressive function against naïve CD4^+^ T cells (16).

Another way IL-27 exerts immunosuppressive effects is through the induction of a potent anti-inflammatory cytokine, IL-10. IL-27 can induce Th1, Th2, and Th17 cells to produce IL-10 in a STAT1/STAT3 dependent manner (79, 80). IL-27 stimulates differentiation of Foxp3^-^ type 1 regulatory T cell (Tr1) that produces IL-10 by increasing the expression of transcriptional factor c-Maf (79, 81–83). IL-27 also contributes to the expansion Tr1 by upregulating autocrine transcription factor aryl hydrocarbon receptor (AhR), which along with c-Maf, transactivates IL10 and IL-21.

In summary, IL-27 was initially thought to be a pro-inflammatory cytokine because of its capacity to induce Th1 differentiation; however, many studies have elucidated its diverse anti-inflammatory effects on CD4^+^ T cell function **(** **Figure 4** **)**.

### CD8^+^ T Cells

IL-27 promotes proliferation of CD8^+^ T cells (84, 85) by inducing transcription factors T-bet and Eomesodermin (EOMES) and increases the production of IFN-γ (84, 85). Additionally, IL-27 is known to increase CD8^+^ T cells’ cytotoxicity by enhancing granzyme B and perforin expression (84, 85). IL-27 also acts as a potent adjuvant activator during vaccination as it promotes the expansion of antigen-specific CD8^+^ T cells. For instance, IL-27 production in DCs following immunization with TLR agonists correlated with induction of antigen-specific CD8^+^ T cells, contribution to bacterial clearance during *Listeria monocytogenes* infection (86–88) **(** **Figure 4** **)**. IL-27/IL-27R signaling have been also reported to be critical to subunit immunization-elicited T cell responses. A loss of IL-27Rα in T cells resulted in a >10-fold reduction in antigen-specific T cell formation in both CD4^+^ and CD8^+^ T cells, and this suppressive effect due to IL-27 deficiency mediated by STAT1 & STAT3 (22). In summary, many reports have showed that IL-27 has important roles in proliferation and activation of CD8^+^ T cells.

### B Cells

IL-27 influences B cells variably depending on the stage of B cell differentiation (35). IL-27 increases proliferation of naïve and germinal center B cells, but not memory B cells (35). In mice, IL-27 promotes Ig class switching to IgG2a in spleen B cells through STAT1/T-bet signaling pathway independently from IFN-γ (89), while IL-27 increases the production of IgG1 by human spleen and cord-blood naïve B cells (90). IL-27 stimulation of human naïve B cells enhances differentiation into a germinal center-like phenotype expressing CD38, CD20, CD95, and CD10 *via* STAT1 activation (91). Also, IL-27 promotes proliferation of B cells *via* induction of Pim-1 (92). In germinal center-driven autoimmunity Roquin^san/san^ lupus mouse model, IL-27Rα^-/-^Roquin^san/san^ mice showed a significantly reduced germinal center B cell numbers, and IgG2a autoantibodies (91). Despite the aforementioned studies, the functional role of IL-27 on B cells remains unclear, and further studies are required **(** **Figure 4** **)**.

## Effect of IL-27 on Infections

### Sepsis

Sepsis is a systemic dysregulated host response caused due to an infection and is associated with acute organ dysfunction and a high mortality rate (93). Traditionally, sepsis was considered to be an initial systemic hyper-inflammatory response to infection. In 1991, the American College of Chest Physicians and the Society of Critical Care Medicine defined sepsis clinically as a systemic inflammatory response syndrome (SIRS) following infection (93, 94). However, recent studies have shown that the pathophysiology of sepsis is more complicated with inflammatory and immunosuppressive responses occurring early and concomitantly during an infection (95, 96). In 2016, the Third International Consensus Definition for Sepsis and Septic Shock (Sepsis-3) redefined sepsis as a life-threatening organ dysfunction following a systematic dysregulated host response to an infection and septic shock as sepsis-associated circulatory, cellular, and metabolic abnormalities (97). Patients with sepsis-induced shock have high serum lactate levels (>18mg/dL), require vasopressors to maintain normal arterial pressure, and unfortunately, remains the leading of cause of death in intensive care units in hospitals with a mortality rate of > 40% (97).

Several studies have reported elevated serum IL-27 levels during sepsis (18, 98–108), suggesting that IL-27 could potentially be a diagnostic biomarker of sepsis (98, 99, 105–107, 109). By using micro array analysis, Wong et al. have showed that EBI3, a subunit of IL-27, had the highest predictive strength for patients with sepsis among the 221 differentially regulated gene probes (107). In a single-center prospective study, ROC curves of critical ill patients with positive blood bacteria cultures yields AUCs of 0.75 for serum IL-27, which was better than AUCs of 0.64 for serum procalcitonin (109).

Interestingly, blockade of IL-27 is beneficial against sepsis (18, 103, 108, 110–112). Mice deficient for the EBI3 subunit of IL-27 were resistant to CLP-induced septic peritonitis, and EBI3^-/-^ mice displayed enhanced neutrophil migration and oxidative burst capacity after CLP (112). However, when we interpret the result of EBI3^-/-^ mice model, we need to be careful and consider the fact that EBI3 can also pair with IL-12p35 to generate the inhibitory cytokine IL-35 (26). In a neonatal murine *E. coli* sepsis model, IL-27Rα^-/-^ mice showed lower levels of TNF-α, IL-1β and IL-6, and improved survival rates. Macrophages from IL-27Rα^-/-^ mice eliminated *E. coli* with increased efficiency *in vitro* and did not induce TNF-α production suggesting that IL-27 indirectly promotes an inflammatory cytokine response during neonatal sepsis by directly compromising control of bacteria that induce the inflammatory response (18). In murine models of polymicrobial sepsis induced by CLP, and endotoxic shock induced by lipopolysaccharide (LPS), blockade of IL-27 with neutralizing anti-IL-27p28 antibodies decreased inflammatory cytokine levels (IL-1β, IL-17 and IFN-γ), and improved the survival rate (110). Similarly, in a murine model of CLP-induced lung inflammation/injury, blockade of IL-27 decreased accumulation of innate cells in the lung and attenuated lung injury, leading to improved survival rate of mice (19).

Several studies have also demonstrated that IL-27 is a beneficial cytokine that can prevent sepsis-induced myocardial dysfunction and death. In an endotoxic shock murine model induced by LPS, blockade of IL-27 by neutralizing anti-IL-27p28 antibody or using IL27Rα^-/-^ mice increased inflammatory cytokines (IL-6, IL-12, TNF-α), and biomarkers of myocardial injury [brain natriuretic peptide (BNP), cardiac troponin (cTn)], suggesting that IL-27 has anti-inflammatory effects and protect against sepsis-induced myocardial dysfunction (101). Yan et al. showed that IL-27p28, alleviates sepsis *via* modulation of cytokine profiles produced by Natural killer-like T cells (NKT cells). In the study, the authors observed that septic mice treated with IL-27p28 encoding plasmid or recombinant IL-27p28 showed improved survival rate, less liver damage, and suppressed lymphocyte apoptosis. Interestingly, NKT cells produced much higher levels of IL-10 and lower levels of inflammatory cytokines (IFN-γ and TNF-α) in IL-27p28 treated septic mice (113).

The relationship between IL-27 genetic polymorphisms (rs153109/-964A and rs17855750/2905) and sepsis has also been studied. No difference in the genotype/allele frequencies were observed between patient with sepsis and healthy controls. However, the rs153109 A allele was overrepresented in patients with severe sepsis/septic shock compared with the patients with mild sepsis. Further, high-risk AA genotype resulted in increased IL-27 levels in isolated PBMCs after LPS stimulation *in vitro*. These data suggest that IL-27 polymorphisms, and subsequently elevated IL-27 levels, do not influence susceptibility to sepsis but exacerbate the severity of sepsis (100).

While it is apparent that IL-27 has clear association with immune dysfunction during sepsis, there is still more to be learned about the exact role of IL-27 in the context of sepsis. The aforementioned studies also indicate that IL-27 effect could be dependent on the infecting bacterial pathogen, the immune status of the host, and the timing of infection. Better understanding of pathogen-specific IL-27 responses during sepsis could be clinically beneficial.

### Bacterial Infections

#### Clostridium difficile

In a murine *Clostridium difficile* colitis infection model, blockade of IL-27 by using IL-27Rα^-/-^ mouse enhanced colonic damage, decreased *C. difficile* clearance and survival rate. Additionally, administration of recombinant IL-27 to WT mice caused increased *C. difficile* clearance, decreased colonic damage, and improved survival rate. Furthermore, recombinant IL-27 treatment in WT mice decreased IL-6 and IL-17 levels, but enhanced production of IL-10 and IFN-γ in the cecal tissue after infection. These results suggests that IL-27 mediates host defense during colitis *C. difficile* infection by downregulating Th17 responses, but upregulating Th1 responses (21).

#### Staphylococcus aureus

In a *S. aureus* pneumonia murine model, IL27Rα^-/-^ mice decreased neutrophil and macrophage recruitment to the infection site compared to the WT mice. However, there was also trending lower bacterial burden in IL27Rα^-/-^ mice in lung compared to WT mice, suggesting that IL-27 influences on recruitment of innate immune cells indirectly reflected its effect on control of *S. aureus* (20). In secondary *S. aureus* pneumonia following influenza infection in mice, IL27Rα^-/-^ mice showed increased levels of IL-17F, decreased levels of IL-10, and exhibited improved bacterial clearance (20). Influenza infection has been reported to inhibit Type 17 immunity, which may lead to increased susceptibility to *S. aureus* pneumonia (114). Interestingly, IL-27 also inhibits Type 17 immunity (69, 71, 115, 116), and so could have synergistically enhanced susceptibility to *S. aureus* infection following influenza infection *via* inhibition of Th17 immunity. The role of IL-27 in mediating immune responses during chronic *S. aureus* infections such as osteomyelitis remains unknown and an area in need of active research.

#### Mycobacterium Tuberculosis

*Mycobacterium tuberculosis* is a unique bacterium known for its ability to survive inside host macrophages by manipulating host defense mechanisms (117–119). It remains a global health threat with an estimated 10.0 million people falling ill annually due to tuberculosis (World Health Organization, Global Tuberculosis Report 2019).

IL-27 affects host immune response against *M. tuberculosis* infection with elevated IL-27 levels reported in granuloma, lung, pleural fluid, and sputum during *M. tuberculosis* infections (6, 120–125). Interestingly, several studies have indicated that macrophages and T cells primarily produce IL-27 in these infections (40, 126). In a murine tuberculosis model, IL-27Rα^-/-^ mice showed improved control of bacterial growth and decreased bacterial burden in lung and spleen on days 30 through 125 following infection (122). Similarly, Holscher et al. showed that IL27Rα^-/-^ mice infected with *M. tuberculosis* showed decreased bacterial loads in the later stages of infections (beyond 42 days) compared to WT mice. Interestingly, IL-27 blockade accelerated mortality in the late infection phase with all mutant mice dying before day 300 of infection due to chronic hyperinflammatory responses. In general, the survival rates of WT mice were higher compared to IL27Rα^-/-^ mice (6). This study suggests that IL-27 both prevents antimycobacterial response and limits chronic hyperinflammatory response in *M. tuberculosis* infection.

*M. tuberculosis* continues to survive within human macrophages through arresting the normal maturation of its phagosome and IL-27 is associated with *Mycobacterium*’s defense against maturation of host phagosome (127). IL-12 treatment combined with anti-IL-27 neutralization decreases pH of the phagosome by increasing phagosomal vacuolar ATPase (V-ATPase) concentration causing enhanced phagosomal acidification and maturation of cathepsin D in the *Mycobacterium*-containing phagosomes (127). Interestingly, treatment of IL-12 with neutralization of IL-27 limited *M. tuberculosis* growth in primary human macrophages and increased inflammatory cytokine (IFN-γ, IL-6 and TNF) production by infected macrophages (128, 129).

*M. tuberculosis* can inhibit apoptosis and induce necrosis of host macrophages, resulting in cell lysis and bacterial spread (119, 130). One study showed that IL-27 subunit EBI3 is associated with this mechanism. In *M. tuberculosis*-treated murine macrophages, EBI3 accumulation increased. Moreover, the intracellular EBI3 inhibits caspase-3 mediated apoptosis in *M. tuberculosis*-treated macrophages (131).

One study showed that stimulation of autophagy in macrophages compels mycobacteria loaded phagosomes to fuse with lysosomes resulting in destruction of the pathogen, thereby suggesting that autophagy is an important host-defense mechanism against *M. tuberculosis* infection (132). IL-27 is believed to inhibit IFN-γ induced autophagy by activating autophagy negative regulatory factors mTOR and Mcl-1 in human macrophages infected with *M. tuberculosis*, thus promoting bacterial intracellular survival (133).

As mentioned, IL-27 induces Th1, Th2, Th17, Treg and Tr1 cells to produce IL-10 (80, 81, 134). Several studies have shown that IL-10 promotes *M. tuberculosis* disease progression in mice (135, 136). Moreira-Teixeira et al. showed that mice deficient in T cell derived IL-10 exhibited a significant reduction in lung *M. tuberculosis* burden during chronic infection. The authors also demonstrated that IL-10 expression in CD4^+^ T cells was partially regulated by IL-27 signaling (137). In summary, *M. tuberculosis* is a unique pathogen due to its ability to survive inside host macrophages, and IL-27 promotes its intracellular survival in many immune cells including long-lived macrophages. Most interestingly, IL-27 prevents chronic hyperinflammatory host response during *M. tuberculosis* infection, a phenomenon that needs to be extensively examined in other chronic bacterial infection setting.

## IL-27-Mediated Immune Homeostasis During Bacterial Infections – A Proposed Mechanism

**Figure 5:**
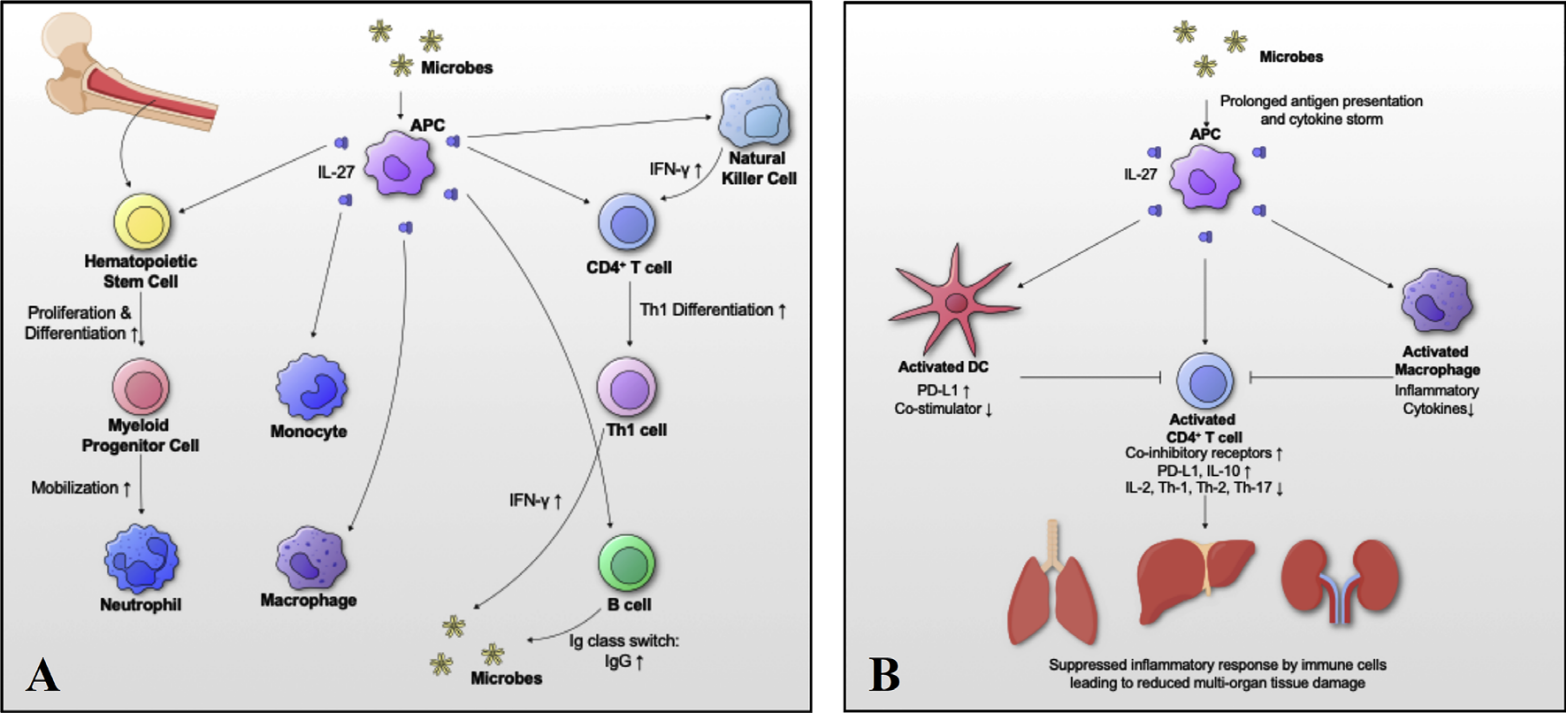
Schematic model of IL-27-mediated immune homeostasis during bacterial infections. To better understand the role of IL-27 signaling in immune responses during bacterial infections, we propose a hypothetical mechanism by which IL-27 regulates activation and return to homeostasis in a time dependent manner. **(A)** At the onset of infection, IL-27 signaling induces myelopoiesis, differentiation and migration of immune cells with Th1 differentiation and IFN-γ production. **(B)** Following the acute response, IL-27 signaling suppresses immune responses to avoid “cytokine storm” and multi-organ failure due to excessive inflammation in vital organs. To control excessive inflammation IL-27: 1) increases expression of co-inhibitory receptors, 2) decreases co-stimulator expression by activated dendritic cells, 3) suppresses production of inflammatory cytokines by activated macrophages and T cells, and 4) promotes production of IL-10 in T cells, all of which suppress inflammatory responses by immune cells and avoid internal organ tissue damage.

The above review has focused on summarizing the immunobiology of IL-27, and discussions on its immunopathology in various bacterial infectious diseases. It has become clear that IL-27 is a crucial immunomodulatory cytokine with both immunostimulatory and immunosuppressive effects. However, further research is needed to understand the exact role of IL-27 in immune response against bacterial infections. Here, we discuss a hypothetical mechanism of how IL-27 regulates immune homeostasis during bacterial infections in a time dependent manner. At the onset of infection, IL-27 promotes immune reaction at the infection site by inducing myelopoiesis, Th1 differentiation and IFN-γ production **(** **Figure 5A** **)**. At later stages of infection, IL-27 suppresses inflammatory responses by immune cells to avoid multi-organ failure due to excessive or sustained inflammation with prolonged antigen presentation and/or cytokine storm. To control inflammation, IL-27 1) increases expression of co-inhibitory receptors, 2) decreases co-stimulator expression by activated dendritic cells, 3) suppresses production of inflammatory cytokines by activated macrophages and T cells, and 4) promotes production of IL-10 in T cells, all of which suppress inflammatory responses by immune cells and avoid internal organ tissue damage **(** **Figure 5B** **)**.

## Concluding Remarks

Bacterial infections remain a serious health burden, leading to significant human morbidity and mortality. With the emergence of multidrug-resistant bacteria, immunotherapies are urgently needed to supplement antibiotic therapies (138–140). Recent studies have evaluated the therapeutic potential of targeting IL-27 during bacterial infections with mixed and conflicting results. The various studies summarized here clearly indicate the need to better understand the context-dependent functions of IL-27 during bacterial infections. As IL-27 has paradoxical pro-inflammatory and anti-inflammatory properties, its administration as a therapeutic treatment may be effective depending on the timing of administration, and the progression of the bacterial infections. Thus, an urgent need exists for better understanding the molecular mechanisms of IL-27 in the development of infectious diseases to target IL-27 in a more successful manner.

## Author Contributions

YM and GM conceptualized and drafted the manuscript with inputs from EM and ES. YM, EM, ES, and GM conceptualized the figures. YM and EM designed and digitized the figures. All authors contributed to the article and approved the submitted version.

## Funding

This work was supported by NIH NIAMS P30 AR069655 pilot grant (GM) with additional support from NIH NIAMS P30 AR069655 (ES) and P50 AR072000 (ES).

## Conflict of Interest

The authors declare that the research was conducted in the absence of any commercial or financial relationships that could be construed as a potential conflict of interest.
